# A Feasibility Study on an Ultra-Brief Intervention for Improving Freshmen’s Emotional Intelligence

**DOI:** 10.3390/jintelligence9030036

**Published:** 2021-07-14

**Authors:** Keith A. Puffer, Kris G. Pence, Abigail E. Ferry

**Affiliations:** 1 Department of Psychology, Indiana Wesleyan University, Marion, IN 46952, USA; 2 Department of Counselor Education and Counseling Psychology, Western Michigan University, Kalamazoo, MI 49464, USA

**Keywords:** emotional intelligence, EI enhancement training, the MSCEIT (Mayer-Salovey-Caruso Emotional Intelligence Test), feasibility study

## Abstract

In 1990, Salovey and Mayer introduced emotional intelligence (EI). Thirty-one years later, a proliferation of interventions to improve people’s EI has taken place. A literature review of studies focused on enhancing the EI of college students revealed a notable gap. When educational material for training sessions included all of the skills in an EI model, researchers usually utilized lengthy durations (i.e., 11–56 h). Few successful investigations employed an ultra-brief (i.e., ≤1 h) approach. The present study examined the feasibility of training using a minimalistic timeframe and a sample of freshmen; their transitional challenges from high school to college mark them as an appropriate target population. Employing a quasi-experimental one-group pretest–posttest design, the recruited participants (n = 75) experienced an ultra-brief intervention highlighting the complete skill-set in the Ability Emotional Intelligence model. Findings from a one-way repeated measures MANOVA indicated improvement transpired in two of four MSCEIT scores (i.e., perception and facilitation). The merit of the present study is delineated using Orsmond and Cohn’s five objectives for feasibility investigations. In addition, implications of the results and possible applications are proposed.

## 1. Introduction

Emotion is fundamental to human experience. This mental process frames and orders cognition, behavior, and social interaction (Johnson 2016). Within the field of emotion science, emotional intelligence (EI) has been a specialty for 31 years. Salovey and Mayer (1990), pioneers in EI, defined the construct as a mental ability, the capacity to engage in abstract reasoning about emotion.

While developments in the theory of EI and its measurement have progressed in the past three decades, interventions for improving EI have proliferated (Kotsou et al. 2019; Zeidner et al. 2012). Authors of meta-analytic studies tend to distinguish the plethora of training approaches by several features (Mattingly and Kraiger 2019). Common criteria include the EI model, educational material, duration of training, and target population.

Training material often, but not always, follows a researcher’s preferred conceptual model of EI (e.g., the ability, trait). The educational content commonly includes one or two specific EI skills or the complete set of skills contained in the selected EI model. Expected length of training time can vary from ultra-brief, brief, to lengthy durations. As for target populations, investigators have worked with several people groups (e.g., parents, school-aged children, professionals) over the past 31 years.

A review of the literature focused on EI enhancement with undergraduates revealed a notable pattern. A majority of investigators selected lengthy timeframes (e.g., 11–56 h) when training content contains all of the skills in the EI model (Hodzic et al. 2017). Few successful studies employed an ultra-brief (i.e., ≤1 h) duration (Kidwell et al. 2015).

The trend is unfortunate. Several advantages with ultra-brief training for freshmen exist. The brevity of the intervention can be an attractive option to educators of freshmen level courses or First-Year Experience classes (NRC 2013; Young 2018). Adding one hour to the curriculum would have more appeal than lengthy durations. After the intervention, freshmen’s improved EI can become a welcomed assistant in adjusting to the different lifestyle of college relative to high school. Further, an intervention in the first year of the university would afford freshmen more developmental time (e.g., about three years) to practice their EI skills toward becoming more deeply embedded into their daily functioning.

Therefore, can an intervention directed toward freshmen using an ultra-brief training duration and covering the complete Ability Emotional Intelligence skillset be effective? If so, what modifications to the intervention need to be pursued in order for it to be relevant and sustainable (Bowen et al. 2009)? The following discussion seeks to answer these questions. We present a rationale for an intervention designed to improve the EI of freshmen using a minimalistic training timeframe. In addition, we delineate the outcomes of the enrichment training session and the merit of this feasibility study.

### 1.1. The Effects of Adative Levels of Emotional Intelligence among Undergraduates

Salovey and Mayer (1990) introduced their model of emotional intelligence (EI) as four information processing skills—perception, facilitation, understanding, and regulation. These hierarchically arranged abilities enable individuals to structure reality from emotional data (Puffer 2011). When EI skills in any model are honed, they can empower humans to thrive.

Evidence in the research literature for adaptive functioning with robust levels of EI among college students is profuse. For mental health fitness, high EI for undergraduates associates with low levels of stress (Thomas et al. 2019), anxiety, and depression (Extremera and Fernandez-Berrocal 2006). Fewer burnout symptoms (i.e., low exhaustion and low cynicism) (Cazan and Nastasa 2015) and angry ruminations (Garcia-Sancho et al. 2016) are also related to high EI scores.

Socially speaking, college students with high emotional intelligence tend to be more interpersonally sensitive, prosocial (Lopes et al. 2005), and culturally intelligent (Putranto et al. 2018). They have less indirect aggression (Garcia-Sancho et al. 2016). High levels of perception of emotion related to lower aggression and high levels of regulation of emotion associated with low physical, verbal, and hostile aggression (Megias et al. 2018). Libbrecht et al. (2014) indicated regulation of emotion also positively correlated to interpersonal academic performance (i.e., active listening and empathy). DiFabio (2015) found high total EI positively associated with perceived social support—aspirations for a variety of relational connections (i.e., acquaintance, romantic, family, and work).

Regarding students’ existential welfare, those with high EI scores often have higher levels of vitality (i.e., high energy and low fatigue) and an increased sense of well-being (Austin et al. 2010). Higher life satisfaction and positive affect correlated with higher EI (Cazan and Nastasa 2015; Extremera and Rey 2016). Huang and Lee (2019) reported EI positively related to affiliative and self-enhancing humor, while it negatively associated with aggressive and self-defeating humor.

Among career development factors, emotionally intelligent college students tend to possess a high level of career decision-making self-efficacy (Jiang 2016). In this regard, they can successfully execute vocational decisions such as assessing their career interests, setting goals, and obtaining pertinent career information. Puffer (2011) reported among female undergraduates, facilitation of emotion positively predicted vocational identity, an indicator of clarity with their career interests and goals.

High emotional intelligence correlates with academic performance and student retention constructs. Total EI positively related to academic success (i.e., the annual average grade) among first-year nursing students (Sharon and Grinberg 2018). Robust levels of EI correlated with low test-anxiety (Ahmadpanah et al. 2016) and lower transition shock when shifting from high school to the university setting (Thomas et al. 2019). Moreover, faculty’s emotional intelligence is also important for undergraduates’ collegiate experience. Frequent student-faculty encounters is one of the key factors in students’ retention. Lillis (2011) discovered undergraduates have fewer attrition plans when appointed to faculty advisors possessing higher EI levels.

Other studies underscore the importance of enhancement training for undergraduates with low EI. For instance, a low level of EI along with low self and body-esteem predicted high social anxiety (Abdollabi and Tallib 2016). Low EI also associated with high trait psychopathy (i.e., cold heartedness, rebellious non-conformity, blaming others) (Fix and Fix 2015). Last, low levels of perception and facilitation of emotion among male students correlated with complicated relationships with peers and substance abuse (Brackett et al. 2004).

### 1.2. An Evaluation of Training Programs for an EI Improvement

Integral to any EI improvement program is the assumption of plasticity (Mayer et al. 1999). Researchers assume EI is alterable, non-resistant to change. In other words, people can learn how to improve their emotional intelligence. This becomes advantageous when improvement to higher levels of EI presents people more opportunities for thriving and flourishing as the aforementioned findings indicated for college students. For instance, a freshman with high total EI often possesses more perceived social support (DiFabio 2015) which can possibly influence a stronger sense of belonging in the new collegiate environment (Walton 2018).

Thirteen years ago, Groves et al. (2008) described the state of training programs for the improvement of emotional intelligence as “incredibly popular” (p. 242). Yet, there was a lack of evidence on effectiveness. Today, popularity remains and empirical support for efficacy continues to be established. The following evaluation examines some of the evidence for effectiveness (i.e., EI trainability) and explains a particular gap (i.e., training duration) in the research literature undergirding the purpose for our feasibility study. Much of our appraisal derives from the findings of 83 studies examined in four meta-analyses (Blanch-Hartigan et al. 2012; Cherry et al. 2012; Hodzic et al. 2017; Kotsou et al. 2019).

#### 1.2.1. The Trainability of EI

Consistently, efforts to improve participants’ EI have been successful (Cherry et al. 2012). In other words, intentional EI education is effective. Mattingly and Kraiger (2019) supported the trainability of EI. They reported moderate effect sizes (ES) for the EI enhancements in their examination of fifty-eight studies; specifically, they found a .45 ES for treatment-control designs and a .61 ES for pre-post designs. Likewise, Hodzic et al. (2017) indicated a moderate effect size (.51 ES) with twenty-four interventions. Blanch-Hartigan et al. (2012) concluded there were substantial improvements in perception of emotion for individuals who participated in training (i.e., 30 programs) relative to those who did not participate. Yet, not all enhancement efforts have proven the malleability of EI; Kotsou et al. (2019) stated only 85% of their forty-six studies revealed significant outcomes with at least one EI skill.

#### 1.2.2. Training Duration

Training duration is a programmatic feature focused on time. Investigators estimate the expected timeframe to improve the EI of a target population. The research literature reveals a diverse range in program duration. This underscores a lack of consensus on how long educational training should transpire to achieve desired outcomes. For instance, Hodzic et al. (2017) concluded longer durations translated into higher efficacy. In their review of 24 EI enhancement studies, 18 h was the mean for training. Yet, brevity proponents demur. Based on their 14 studies, Cherry et al. (2012) contended “best effects” from interventions transpired over a short timeframe (p. 11). Blanch-Hartigan et al. (2012) argued increases in timeframes for training sessions did not improve efficacy. Six hours was the average duration in their meta-analysis of 30 studies.

From the previously mentioned four meta-analyses, we identified the three ranges of time, ultra-brief (≤1 h), brief (2–10 h), and lengthy (11–56 h) from 83 of the 114 investigations. A frequency count revealed, 12% of the studies were ultra-brief (i.e., 10/83 articles), 25% were brief (i.e., 21/83 articles), and 63% were lengthy (i.e., 52/83 articles). We utilized only 73% of the articles, because these authors had clearly identified information necessary for the purposes of our study (i.e., EI model, educational content, training duration, and target population).

### 1.3. A Gap in the Research Literature and an Alternative

Resolution of the duration debate will most likely transpire in the distant future. Meanwhile, when the literature review narrows to only undergraduates, the notable gap becomes apparent again. Duration is also not kept ultra-brief (i.e., ≤1 h) when researchers train college students on the complete skill-set of an EI model. More specifically, 33 of the 83 aforementioned articles (i.e., 40%) selected college students as the target population. Ten of the 33 articles had an ultra-brief duration; only four of the ten studies had training material using the complete set of EI skills from the researchers’ preferred EI model. Continuing this literature analysis further, three of these four investigations had a report with significant findings. Yet, the three articles had methodological nuances that differentiate them from the present study. One study trained college students with the EI abilities in separate groups, one skill per group. The other two investigations had samples comprised of older undergraduates.

What is unclear in the research literature is whether a minimalistic intervention with the complete skill-set as training content for freshmen can be effective. Ultra-brief interventions have great potential with this target population. First, it is an approach that can easily be inserted into the curriculum of typical freshmen classes (e.g., General Psychology, Principles of Sociology) or the First-Year Experience courses (NRC 2013; Young 2018). FYEs have become increasingly popular in higher education within the United States since the 1970s. According to a 2017 survey, more than 70% of institutions offered a first-year seminar/class to assist freshmen socially and academically (Young 2018). Yet, the course curriculum tends to be crowded (Garner 2018). Lengthy EI enhancement programs would be difficult to insert into the curriculum; these durations would compete with the plethora of other topics (e.g., practical study habits, time management, campus polices) in a semester long course. Second, an enhanced EI can become an essential aid in coping with the common transitional challenges from high school to the university setting (Walton 2018). Third, first-year students, exposed to an EI improvement early in their academic journey, potentially have more time (e.g., approximately three years) to develop their EI skills within an academic zeitgeist that positively prompts its constituents toward life preparedness (Oberst et al. 2009).

Therefore, the intent of this feasibility study was to assess the appropriateness of an ultra-brief EI intervention in a freshmen general education course. We pursued the following research questions:

- Can an EI enhancement training session including the complete skill-set for Ability Emotional Intelligence (AEI), functioning under an ultra-brief time constraint (i.e., ≤1 h), and targeting freshmen be successfully implemented?
- If so, how will the recruited participants respond to this intervention (Orsmond and Cohn 2015)? More specifically, what modifications to the intervention will be necessary for it to be sustainable (Bowen et al. 2009)?

In the present investigation, a prelude to other preliminary studies before the main study (Tickle-Degnen 2013), we had two outcome expectations.

**Hypothesis** **1.** *A 55-min training duration will be successful.*

○ *The effective 45-min duration of training by Kidwell et al. (2015) and the 30-min interventions of deTurck et al. (1990) and deTurck (1991) support the potentiality in an ultra-brief duration. Yet, the former used different sample groups, one for each EI skill; the other two studies employed training content that focused on only one EI skill.*

**Hypothesis** **2.** *Participants’ AEI, as measured by the MSCEIT (Mayer-Salovey-Caruso Emotional Intelligence Test Mayer et al. 2002), will be improved after the intervention.*

○ *Specifically, the posttest means of Perception, Facilitation, Understanding, and Regulation will be higher than the pretest means at a level of significance of .05.*
○ *The meta-analysis of Hodzic et al. (2017) substantiates this expectation.*

## 2. Materials and Methods

### 2.1. Participants

Participants were comprised of first-year college students enrolled in General Psychology courses from a Midwestern university. The 75 freshmen (57% female; 43% male) who volunteered averaged 18.7 years of age. The age range was 17–20. The upper and lower limits of ages, 17 and 20 year-olds (n = 5), were retained based on the understanding that all participating undergraduates were enrolled in their first-year at the university.

The college students declared 16 academic majors. The most prevalent majors comprised 64% of the sample: business (19%), education (17%), nursing (15%), and pre-declared [i.e., undecided] (13%). The remaining 36% included exercise science (7%); criminal justice, creative arts therapy, Christian ministries, and sports management (each major was at 4%); communications, general studies, and English (each major was at 2.7%); and psychology, political science, pre-medicine, and community development (each one was at 1.27%). Further, regarding ethnicity, most volunteers were European American (89%). Three percent of the respondents self-identified as Latino/Hispanic, 3% as Asian American, 1% as African American, and 4% as other.

We selected freshmen as a target population, because the proposed intervention has significant relevancy to them (Orsmond and Cohn 2015). An improved EI can assist their coping strategies as they experience numerous transitional challenges from high school to the university setting (Yeager et al. 2016). For instance, freshmen quickly realize changes in demands and structure. Academic expectations have heightened, yet there is less academic structure; social autonomy has increased, but there is less accountability (Martin 2015). Often, personal limits in their maturity level are exposed (e.g., poor study habits, high neuroticism) (Clark 2005; Stein 2009). Hence, learning how to manage persistent confusion, unfamiliar negative emotions, and depression become formidable tasks (Gall et al. 2000; Hailikari et al. 2016; Porteous and Machin 2018).

In addition, incoming undergraduates grapple with issues of belonging, ability, and purpose (Walton 2018). There is a nagging uncertainty about belonging. Will peers respect, value, and include them? Can they attach to a community of friends? They question their academic ability to perform in the university setting. Will they handle the new kind of academic rigor and lifestyle changes? Will they succeed at this level of learning and adjust to social differences? College students also muse about academic purpose. Does the university experience matter? Do they have sufficient motivation to tackle academic requirements? If tasks such as homework become boring or even difficult, should they stop trying?

Last, recent statistics on the mental health of college students related to the effects of the pandemic underscore the importance of offering an intervention to enhance freshmen’s EI. According to Wang et al. (2020), 48% of their sample (n = 2031) had a moderate to severe level of depression and 39% possessed similar levels for anxiety. Further, the larger part of the sample (71%) reported higher stress levels during this season with COVID-19. Relative to the other classes, freshmen (13% of the sample) had the highest scores in depression and tied with sophomore for the highest levels of anxiety.

### 2.2. Procedure

The present study lasted two years; five iterations of the intervention transpired. We pursued the assistance of professors of General Psychology sections to recruit freshmen. Research participation is a common means for students to improve their grade in the class. The instructors who granted us permission were willing to incentivize their students with extra credit points for participation in the project. The range of extra credit was 10–15 points; educators add the earned points to the participants’ summative total used to determine the final letter grade in the course.

To introduce the study, the professors allowed 10–15 min at the start of one of their classes. Any college student in attendance received an informed consent form containing the purposes of the research, risks in the study, the promise of confidentiality, and the freedom to terminate along with a request for demographic (i.e., personal) information (e.g., age, major, gender, race/ethnicity). This complied with the protocol of the university’s Institutional Review Board. We, then, verbally processed the content inviting interested persons to fill out the requested items of information.

In general, our intervention process encompassed three activities. Participants were to complete an online, performance-based emotional intelligence (EI) test twice and attend a lecture/discussion on emotional functioning (the intervention) scheduled between the test administrations. Interested college students (n = 81) also received an instruction sheet for accessing the EI test, the MSCEIT (Mayer et al. 2002); they were also informed of a one-week deadline to complete the first administration.

Approximately one week later, we returned to engage the freshmen in the emotional functioning presentation. The same researcher led each intervention lecture. At the close, participants who had completed the first administration of the MSCEIT (n = 75) received a second instruction sheet on how to access the same MSCEIT online test again using a different account. The second administration also had a one-week deadline, an effort to minimize possible history and maturation effects (Wang and Morgan 2010).

### 2.3. The Intervention

The intervention was a 55-min training session. For the educational material, we developed a program derived from several different authors who specialize in emotion science, in general, and who favor the Ability Emotional Intelligence (AEI) specifically. The presenter utilized PowerPoint (i.e., 21 slides) and a seven-page handout (i.e., a replicate of the information on the slides and an assessment). The content commenced with the undergraduates listing five pleasant and unpleasant emotions and sharing their selections to the whole class. When the discussion completed, definitions of and purposes for emotions, followed. Then, we explained and illustrated each skill within in the AEI model—perception, facilitation, understanding, and regulation. The session closed with an ungraded quiz containing 36 questions—true/false (10) and fill-in the blank (26). Participants answered questions using the handout and in collaboration with classmates. We (the presenter and college students) reviewed the educational material by sharing the correct answers to the quiz. Completed handouts were collected at the end of the class period to analyze participants’ engagement during the intervention (Orsmond and Cohn 2015).

Notably, the intervention followed some efficacious training practices mentioned in the meta-analyses of EI enhancements. Mattingly and Kraiger (2019) noted the importance of discussion and skill practice. In our educational session, bi-directional engagement was the intention. We purposely segmented the timeframe to include opportunity for students’ questions, their responses to the presenter’s questions, and solicitation of their ideas and opinions on the presented topics. For instance, students read aloud the descriptions of 21 emotions and processed the merit of the content during the section on understanding emotion. Moreover, some active rehearsal of material transpired. The undergraduates practiced when connecting facial expressions with certain emotions and matching definitional statements of feeling with the appropriate emotion during the quiz (i.e., fill-in-the blanks).

Hodzic et al. (2017) underscored the importance of balancing theory with activities. We disseminated theory via the definitions of, purposes for, and examples of each AEI ability. For example, after explaining regulation of emotion, we illustrated the ability with examples such as self-soothing, mood maintenance, and mood repair.

### 2.4. Measure

The measure for EI was the online version of the Mayer-Salovey-Caruso Emotional Intelligence Test (MSCEIT) with expert scoring (Mayer et al. 2002). The MSCEIT comprises 141 ability-based questions. The items test the competence of respondents in processing and performing tasks, an approach consistent with mental ability testing. The authors of the MSCEIT purposely eschewed the self-report format where respondents indicate their beliefs (i.e., self-perceptions) about skill level (Mayer et al. 2016).

There are eight different sections or kinds of tasks in the MSCEIT. Examples include facial recognition, facilitation tasks (e.g., how mood assists problem solving), change tasks (e.g., how emotions transition to another affect), and emotion management tasks (e.g., mood maintenance). Most respondents can complete this version of the MSCEIT in 30–45 min; yet, no time limit restricts their test taking.

Seven scores are generated from the MSCEIT; these include perception, facilitation, understanding, regulation, total EI along with experiential and strategic EI (i.e., two area scores). For the purposes of this study, we used only four of the seven scales—the four abilities. Concerning psychometric information, Mayer et al. (2012) mentioned the following estimates for reliability: perception—.92; facilitation—.81; understanding—.80; and regulation—.84. Further, Mayer et al. (2001) delineated an exploratory factor analysis supporting the factor solutions for the seven scales in the MSCEIT (i.e., the four abilities, total EI, and area EI).

Regarding the different functions in each of the four abilities, the following descriptions provide specific details.

- *Perception of Emotion (PE)*
  ○ PE is the ability to detect, appraise, and express emotion. Detection entails distinguishing and naming affect within self and others. Appraisal refers to people discriminating authentic and inauthentic expressions of affect (Mayer et al. 2016). Expression denotes persons articulating emotional observations of self and others; they symbolize (i.e., put into words) affective signals (Caruso and Salovey 2004).
- *Facilitation of Emotion (FE)*
  ○ FE involves harnessing emotion to enable a variety of cognitive functions (e.g., problem-finding, problem-solving) (Salovey et al. 2008). People use affect while resolving difficulties, arrange their thoughts by using emotions, and trigger their emotions to make important decisions (Mayer et al. 2016). FE also empowers individuals to empathize with others, feeling the emotions of others (Caruso and Salovey 2004).
- *Understanding of Emotion (UE)*
  ○ UE entails people comprehending the vast emotional language and appreciating the elaborate relationships between emotions (Salovey et al. 2008). It involves possessing a robust emotional lexicon and knowledge base, recognizing the interconnections between emotions, identifying triggers for their emotions, interpreting complicated and even contradictory states of feeling, and anticipating emotional transitions (Mayer et al. 2016).
- *Regulation of Emotion (RE)*
  ○ RE entails individuals monitoring and reflecting on feelings—personal ones and others. It also includes engaging, prolonging, and detaching from favorable and unfavorable emotions, inspiring others via emotions, and energizing self for adaptive living (Mayer et al. 2016).

## 3. Results

### 3.1. Research Design

For this feasibility study, we utilized a quasi-experimental one-group pretest–posttest design. Wang and Morgan (2010) described the pre-experimental approach as marking two observations. The first (pre-test) provides a baseline that precedes the intervention with the second observation (post-test) evidencing changes in the target variables (APA 2020).

Selection rationale were two-fold. First, the pre/post-test design was a response to the notable gap in the research literature. None of the previously mentioned investigations (i.e., 83 studies) attempted or successfully executed an intervention similar to our proposed approach—an ultra-brief training session with freshmen using the complete skill-set of the Ability Emotional Intelligence model. Ma et al. (2019) highlighted this design type as being appropriate to offer “tentative insights about an intervention” (p. 975); Malboeuf-Hurtubise et al. (2017) underscored its capability to analyze “the impact of a novel intervention” (p. 475). Second, a one-group instead of a two-group approach became necessary. Due to unforeseen complications (i.e., timing, corruption of participants), an appropriate control condition was not established when this study commenced.

### 3.2. Preliminary Analyses

Before testing our hypotheses, we conducted three preliminary analyses (Supplementary Materials). Using SPSS (v. 24), we checked for possible effects on participants’ EI due to age, gender, and academic major (IBM Corp 2016). First, there is some evidence in the literature regarding the influence of age. Cabello et al. (2016) reported middle-aged persons having higher EI scores than younger and elderly adults (i.e., an inverted-U curve). Mayer et al. (2002) indicated older participants (e.g., ages 25–34) tend to have higher EI scores than younger adults (e.g., ages 18–24). To detect possible age effects, we ran Pearson product-moment correlations. The findings revealed no statistically significant relationships between age and the eight EI scores (see Table 1). Possibly, comparisons of people near the same age range (i.e., 17–20 years old) result in more similarities in AEI scores than differences.

Second, reporting gender effects with EI, Mayer et al. (2002) indicated females tend to have higher scores on all of the MSCEIT variables. Yet, in this study, we conducted independent samples T-tests and did not detect statistically significant gender differences among the eight emotional intelligence scores (see Table 2). Possibly, the absence of male-female distinctions was due in part to the youthfulness of our participants. Further, Fischer et al. (2018) partially corroborate our findings. From a large community sample (n > 5000), they discovered no support for the emotional sensitivity hypothesis, women having higher discernment with subtle emotional cues.

Third, pertaining to participants’ majors, our concern was the potential effects that the type of academic programs may have on participants’ EI results. Certain majors in the academy (e.g., human service ones) might have attracted or required persons with more emotional savvy. Of the sixteen declared majors, four comprised the majority (64%) of the freshmen. Relative to the other 12, these four majors had a sufficient number of participants per major to run an ANOVA. Hence, we recoded the major variable into four groups—business, education, nursing, and pre-declared. Conducting an ANOVA, we discovered no statistically significant effects of major on the eight EI scores at the *p* < .05 for the four categories of major (see Table 3).

### 3.3. One-Way Repeated Measures Multivariate Analysis of Variance and Effect Sizes

With preliminary analyses showing no statistically significant relationship between participants’ EI and their age, gender, or academic major, we analyzed pre/post scores for the EI subscales using a one-way repeated measures multivariate analysis of variance (MANOVA). The advantage of MANOVA is that it allows the researcher to examine whether there is a statistically significant difference in the dependent variables using a linear combination while also providing a univariate test of each dependent variable (Pallant 2020). Relative to other procedures (e.g., paired sample T-test), MANOVA has the advantage of protecting against inflated Type I error.

There are a number of assumptions with MANOVA; our analyses of sampling size, outliers, linearity, and multicollinearity revealed no serious violations. Normality was evident in six of the eight EI variables. However, the distribution of pretest and posttest regulation (RE a and b) scores had a moderate negative skew. We conducted a square root transformation and the distributions normalized (Tabachnick and Fidell 2019).

In Table 4, we present descriptive statistics for the four EI subscales. Notable trends pertain to the means of the eight EI scores. For the pretest, the means range from 101 to 115. According to Mayer et al. (2002), there are seven qualitative ranges for AEI scores. Pertinent ones for this study include 100–109—high average score and 110–119—competent. Hence, the mean for perception of emotion (PE) is in the high average level, while facilitation of emotion (FE), understanding emotion (UE), and regulation of emotion (RE) are in the competent level. For the posttests, the means range from 112–117. PE is still high average (i.e., the higher end of the range), while FE, UE, and RE still fall within the competent range. Caruso (2004) defined competency as ‘‘sufficient skill to perform with some degree of success’’ (p. 6).

Results from the one-way repeated measures MANOVA are presented in Table 5. The findings show a statistically significant effect for time (pretest to posttest) for the combined dependent variable, F(4, 70) = 5.52, *p* < .001, Wilks’ lambda = .760, partial eta squared = .24. Univariate results reveal statistically significant differences in two of the four EI scales, perception and facilitation of emotion.

Furthermore, effect sizes (ES) in Table 5 indicate the magnitude of the difference between the time variables and the pre/post univariate means. Cohen (1992) characterized a partial eta squared of 0.01 as a small ES, .06 as a moderate ES, and .14 as a large ES. Notably, the proportions of variance in perception (23%) and facilitation (5%) explained by time were notably different (Pallant 2020).

### 3.4. Assessment of Feasibility Objectives

To examine the merit of this feasibility study in a comprehensive manner, we evaluated our research process via the five objectives developed by Orsmond and Cohn (2015). Their five goals delineate key components that mark the efficacy of a feasibility investigation. Each of the five objectives answer a specific question. Those inquiries include: (1) Were our participants appropriately recruited; (2) Were our data collection methods and outcome measures suitable; (3) Were our procedures and intervention relevant to our participants; (4) Was the research team equipped with important resources and experience to direct the study and intervention; and (5) Was the intervention successful with the identified population.

#### 3.4.1. Recruitment of Participants

Orsmond and Cohn (2015) discussed participant recruitment in terms of accessibility and criteria for eligibility. Our target population was freshmen enrolled in General Psychology. The selected university for recruitment offered 10 sections of this general education course with a capacity of 40 per class in the fall; spring scheduling included five sections with the same capacity.

Our efforts for recruitment covered one fall and two spring semesters. Although not all students are freshmen in General Psychology, there were sufficient eligible participants available to us (i.e., n > 600). Research participation is a popular means employed by educators of General Psychology for extra credit.

Our eligibility criterion was simple. Participants needed to be in their first year of college. Ineligible people were older undergraduates (e.g., anyone >20 years old, a sophomore, etc.) or a high school student taking General Psychology in escrow. We eliminated four people who were 24 years old and retained seven students who were either 17 or 20. For the latter, the class professors informed us that these individuals were enrolled at the university for the first time.

The relevancy of our EI intervention to freshmen is substantiated in the research literature covering transitional challenges (e.g., a sense of belonging, mental health) (Martin 2015; Walton 2018). To estimate their need for the training, we used the recruitment dates for each iteration of the intervention. Twenty-five percent of the sample had one month of experience in university life. The remaining (75%) had five to six months of experience. For more precise evidence of need, we suggest that in subsequent preliminary studies researchers add a brief questionnaire assessing freshmen’s sense of belonging, academic ability, and academic purpose along with anxiety/stress and depression levels. This self-report and rapid assessment can include a Likert scale of 1 (low level) to 5 (high level) for each item; it could also be added to the backside of the consent form used during recruitment.

Although freshmen were accessible, this study had only five iterations of the intervention over two years. Our recruitment procedures appeared appropriate and sufficient; yet, the pace of recruitment turned out to be inefficient. Of 20 available sections, only five were opened for recruitment—a 75% refusal rate. The faculty for General Psychology mentioned being interested in and supportive of the research topic. The major deterrent was the request for an in-class intervention instead of an out-of-class one. In other words, we were asking for a whole class period (i.e., 55 min) in addition to the 10–15 min to recruit. Many educators indicated not having enough time in their semester schedule to include our study. Possible solutions include requesting the whole class period in advance (e.g., the previous semester) before instructors/professors establish their schedule.

The in-class approach was an intentional selection; we wanted to assess whether the intervention could be delivered under specific time constraints and a limited budget (Bowen et al. 2009). When we look to the future, when modifications to the intervention are completed, we foresee a whole freshmen class via a general education class or a First-Year Experience course having easy access to and benefit from an EI enhancement training session with minimal expense. In contrast, the ‘out of class’ approach had less appealing features—not a real-life classroom setting, potentially inconvenient to participants, time-consuming, and more expensive. However, these perceptions are unstudied. The present feasibility study was limited by the number of participants who could enter the study at one time (i.e., 40 freshmen) and by an elongated research process (i.e., two years). Possibly, an increase in preliminary phases could establish the best context. Another feasibility study could test our intervention in the out-of-class context followed by a pilot study using the in-class context again.

#### 3.4.2. Data Collection and Outcome Measures

The second objective examines the procedures utilized in collecting data from participants and the suitability of the outcome instrument. In our study, the freshmen engaged in four activities. They filled-out the consent form requesting five pieces of personal information (e.g., age, gender, and ethnicity). They completed two administrations of the online version of the MSCEIT that contained 141 performance-based questions. Last, they wrote out answers on the intervention handout that solicited emotional preferences (i.e., pleasant and unpleasant ones) and assessed their retention of the presented information (i.e., a quiz). In general, these procedures do not appear to be burdensome; they were uncomplicated in nature and made convenient for them. For instance, the freshmen completed the consent form during a regular class meeting; the completion of the handout transpired during the intervention; and they could take the administrations of the MSCEIT online at a time and location of their choosing.

The outcome measure was the MSCEIT (Mayer et al. 2002). As previously mentioned, this instrument has sufficient psychometrics (Mayer et al. 2012). Further, the authors developed the MSCEIT for people 17 years of age and older. They calculated its readability to be a North American 8th grade reading level. Since the mean age of our sample is 18.7 years old and the freshmen’s MSCEIT scores were in the expected range, we assumed the freshmen adequately comprehended the questions in this EI measure and demonstrated the needed capability to complete our data requests.

Although we found the MSCEIT to be a suitable outcome measure, we suggest four additional outcome measures for inclusion in subsequent preliminary studies. Most likely these would be administered prior to and possibly after the study. First is a social desirability assessment that can alert researchers to participants’ impression management tendencies that may negatively influence their approach to both MSCEIT administrations. The shortened version of the Marlow-Crowne Social Desirability Scales (SDS) with 13 items can be a simple, efficient, and economical means of assessment (Reynolds 1982). Second is a processing speed appraisal; investigators would benefit from knowing how well the freshmen receive, comprehend, and respond to information. Possibly, the Symbol Search (SS) subtest in the WAIS-4 would be a suitable instrument (Wechsler 2008). Third is an evaluation of participants’ personality traits that may influence their engagement with the intervention. Rossen and Kranzler (2009) reported the total EI in the MSCEIT positively related, albeit a small correlation, to openness, agreeableness, and neuroticism. College students with high scores on these trait factors might be more receptive to the intervention. The short version of the five-factor model (BFI-S; Soto and John 2017) with 15 items can be a brief measurement of these specific personality features. Last is an evaluation of functional outcomes from the intervention, a pre- and post-study estimation. Possibly, researchers secure academic achievement, mental health, and social relational data prior to the study as another means to register evidence of need for the intervention and after the study to ascertain long-term benefits of an enhanced EI. Specifically, they could garner the freshmen’s GPA (i.e., for second semester students), psychological well-being via the 43-items of the Scales of Psychological Well-Being-Short Form (SPWB-SF; Ryff 1989), and social belonging via the 25-items of the Inventory of Parent and Peer Attachment (IPPA; Armsden and Greenberg 1987).

New questions and challenges rise with each additional outcome measure. What impact does the added financial costs have upon the research budget? Will additional questions (i.e., approximately 100) burden participants? Yet, the benefits could outweigh the negatives. More thoroughly screened participants may result. Possibly, future researchers can create brief versions for some of the aforementioned variables (e.g., rapid assessments with five-six items per variable using a Likert scale).

#### 3.4.3. Intervention and Participants

The third objective of Orsmond and Cohn (2015) inquires about the relevancy of our procedures and intervention as perceived by the participants. Initially, 81 college students indicated interest in our study; at the end of the second MSCEIT administration, 75 freshmen had persisted. This completion rate indicates the majority of individuals attracted to the study became ‘finishers.’ Of the remaining six undergraduates, either they were 24 years old and eliminated or they filled out the consent form, completed the first administration of the MSCEIT, and did not re-take the MSCEIT. However, it is important to note that our sample emerged out of a pool of 184 students. With 75 people participating, there was only a 41% acceptance rate. Possibly, the subject of our research was not interesting or the need for extra-credit was lower than expected.

When combined, the research tasks in our study required a three-hour time investment. Completion of the consent form took about 15 min; the intervention lasted 55 min; both administrations of the MSCEIT required approximately 80 min. These activities appear to be minimal intrusions in participants’ daily schedule. Further, we intentionally designed the recruitment effort, the intervention, and MSCEIT administrations to be as convenient as possible. The freshmen completed the consent form and engaged in the intervention during a regular class period. Each student had sufficient time to disclose personal information and provide answers to questions on the handout used during the intervention. Online administrations of the MSCEIT afforded the opportunity for students to select a time and location that best suited them. In addition, there are no time limits when taking the MSCEIT. Participants were able to take as much time as needed. The usual completion time is between 30–45 min. (Mayer et al. 2002).

Regarding the freshmen’s engagement in the intervention, several indicators reveal a moderate level of adherence to the research process. First, we calculated the time interval between the day of recruitment and completion of the first administration of the MSCEIT. The average was 15 days; variations occurred among the different iterations of the intervention. The shortest was one day, while the longest 29 days. Second, the average duration from the intervention to the second administration of the MSCEIT was four days; the range was 1–7 days. This underscores that participants complied with the one-week limit mentioned at the end of the intervention. Third, the mean number of days for the time-interval between the two MSCEIT administrations turned out to be 12 days with a range of 6–19 days. Dutil et al. (2017) indicated 14 days as the frequently recommended interval. For future preliminary investigations, we suggest a longer interval, something closer to the average retesting time of three weeks used with the Wechsler (Estevis et al. 2012). A longer interval will help reduce practice effects, yet directing participants toward this timeframe will require creativity. Possibly, future researchers could monitor when the last participant took the MSCEIT and schedule the intervention three weeks from that date. However, last minute scheduling may be problematic (e.g., General Psychology professors). Fourth, on the intervention handout we requested participants to list five emotions that they enjoy and do not enjoy. The average total was eight emotions, four pleasant and four unpleasant affect. Finally, we checked compliance with the quiz located at the back of the handout. There were four kinds of questions on this brief assessment: (1) Read a statement and fill in the blank with the correct emotion (e.g., a reaction to a loss is …); (2) Identify the emotion displayed on four faces (i.e., facial recognition); (3) Fill in the blank using a ‘word or phrase bank;’ and (4) True/false questions. Based on our analysis, 97% of the college students completed the quiz. For future studies, we suggest altering the quiz questions into more exercise or active type of inquiries, similar to the facial recognition item. Our recommendation is to have participants do something with the information instead of only recalling what we presented in the training session. For instance, a respondent might have to recognize feelings in this manner: “If you observed a person crying and unhappy, what emotion is this most likely to be?” We are not suggesting making replicas of the MSCEIT items; this proposal is about engaging the emotional functioning material in more of a real-life manner. Furthermore, other possible observations during the intervention for tracking participant engagement include noting how many students asked questions, volunteered to read the content on the PowerPoint slides, and collaborated with peers during the quiz.

Last, we did not randomize the participants. Their enrolled class remained their assigned group. If future preliminary studies include an out-of-class option for the intervention, randomization could be easily inserted into the research process.

#### 3.4.4. Research Team

The fourth objective of Orsmond and Cohn (2015) centers on the investigative experience of and available resources to the research team. In this feasibility study, the investigative partners, collectively, have experience with both small- and large-scale projects. They have led or assisted other investigations on this subject and outcome measure (i.e., emotional intelligence, the MSCEIT) and other topics.

As for resources, the lead author secured a research discount from the publisher of the MSCEIT and a scholarship from the Scholarship Council of his university to cover the remaining costs of the outcome measure. Research assistants who were familiar with data entry and statistical procedures were hired using his research budget. No equipment was necessary in the study.

Ethically speaking, we adhered to the university’s IRB protocol. Participants learned about the projects’ purpose and risks, both visually via the consent form and verbally during the recruitment meeting. Their identity remained confidential; participants were free to terminate; and no one was manipulated to persist. Over the two-year span of this project, no adverse effects from recruitment, the intervention, or MSCEIT administrations transpired.

#### 3.4.5. Promise of the Intervention

Orsmond and Cohn’s (2015) final objective examines our estimation of success for the ultra-brief intervention with participants. Was the proposed effect from the interplay between the intervention and outcome measure successful? If not, what modifications are needed?

To ascertain success for our ultra-brief intervention with freshmen, we analyzed the pre- and post-scores of their four MSCEIT scales followed by effect size calculations. Statistically significant increases transpired in two of four outcome measures (i.e., perception and facilitation) with effect sizes ranging from small to large. These findings are promising yet, we have three concerns. First, the lack of a control group makes us hesitant. Observed changes may not be due solely to our intervention (Malboeuf-Hurtubise et al. 2017). Second, understanding (UE) and regulation of emotion (RE) did not change in the expected direction. Past research findings revealed these skills being enhanced with a brief intervention timeframe (Kidwell et al. 2015). Third, the small effect size for facilitation of emotion (FE) questions the effectiveness of the present content for this ability.

In a re-examination of the educational material, we discovered two main gaps. It is possible the importance of an improved EI was lost on our participants. Recent research showing positive correlations between robust levels of EI and mental health fitness and social savvy need inclusion (Austin et al. 2010; Cazan and Nastasa 2015; Lopes et al. 2005; Thomas et al. 2019). Further, the material in our intervention tended to be more passive and static in nature. Instead, the general information about the four AEI skills needs to be accompanied with illustrations that prompts participants to grasp how the abilities operate in action. For instance, with the material for understanding emotion, we need to address how affect transition (changes) and combine (blends). Hence, the content for the PowerPoint slides, handout, and the quiz should focus more on the possible benefits from EI enhancement and real-life applications, emotions experienced in daily life.

Furthermore, concerning the future preliminary studies, we believe a second feasibility study followed by a pilot study are plausible next steps prior to a main study. For the next feasibility study, we review the previously mentioned suggestions. The context of the intervention is switched to an out-of-class scenario. Researchers create a control group with all participants randomized. They maintain the ultra-brief duration at 55 min and develop the aforementioned modifications to the content on the PowerPoint slides, handout, and quiz. Further, after recruitment and prior to the first administration of the MSCEIT, investigators obtain evidence of need. They start with previously proposed changes to the consent form (i.e., the rapid assessment of freshmen’s sense of belonging, academic ability and purpose using a Likert scale) and additional outcome measures (i.e., social desirability, processing speed, personality traits, functional outcomes—mental health or well-being) are disseminated and collected. The timeframe between the two administrations of the MSCEIT is directed to achieve a three-week retesting interval. Monitoring of participants’ engagement during the intervention is increased; calculations of the listing of emotions and quiz completion are joined with counts of how many freshmen ask questions and volunteer to read presentation material during the presentation. Qualitative feedback of participants’ experience with the intervention is ascertained the day after the presentation. After this second feasibility investigation, we suggest a pilot study is conducted with an in-class context. Insight garnered from the two previous feasibility studies can guide how participants will be recruited and how much screening is necessary, how the intervention’s message will be presented, how participants’ participation will be observed during the intervention, how the re-testing interval with the MSCEIT will be ensured, and how the control group will be run.

Finally, the partial success of our findings buoys our confidence in an ultra-brief EI enhancement. The imagined effects of our novel intervention in the lives of freshmen, in our minds, warrants further development. The present study identified gaps; other parameters needed to make the intervention relevant and sustainable (Bowen et al. 2009).

## 4. Discussion

As emotional beings, humans experience hundreds of feelings per day (Goleman 2019). An enhanced emotional intelligence (EI) can conceivably empower individuals toward adaptive living. Within the past 31 years, researchers have been testing interventions with a variety of durations to improve the EI of individuals (Groves et al. 2008). The present feasibility study garnered a sample of freshmen to investigate the effectiveness of an ultra-brief (i.e., 55-min) training session intended to enhance their EI. Such a development has the potential to embolden them when encountering well-known transitional challenges in the first-year of the collegiate experience. Implications of and applications from the findings in this study merit further discussion.

### 4.1. Implications

A few implications from the upgrades pertaining to perception (PE) and facilitation (FE) are discernable. First, the two enhancements in this study corroborate previous research. Several researchers reported improvements to one of the variables or both subscales due to their respective interventions. Regarding the PE upgrade, Herpertz et al. (2016); Kassin and Fong (1999); and deTurck (1991) indicated similar positive outcomes. Yet, their research objective focused solely on this one EI skill (i.e., a partial skill set). Moreover, Cejudo and Latorre (2015); Crombie et al. (2011); and Di Fabio and Kenny (2011) discussed enhancement in PE. In each study, the MSCEIT was the measure of EI; but, in contrast to our study, they selected lengthy durations. Kidwell et al. (2015) had an ultra-brief duration (i.e., 45 min) with their PE upgrade. However, they separated the four Ability Emotional Intelligence (AEI) skills; a different sample was trained for each ability. Concerning the FE upgrade, Cejudo and Latorre, Crombie et al., DiFabio, and Kenny, and Kidwell et al. also found enhancement possible.

Second, the enhancements in this study offer some support for our hypotheses. In the first hypothesis, we predicted:

*A 55-min training duration will be successful.*

In the second hypothesis, we mentioned:

*Participants’ AEI, as measured by the MSCEIT (Mayer-Salovey-Caruso Emotional Intelligence Test; Mayer et al. 2002) will be improved after the intervention. Specifically, the posttest means of Perception, Facilitation, Understanding, and Regulation will be higher than the pretest means at a level of significance of .05.*

Only two of the four MSCEIT scores improved as predicted; understanding (UE) and regulation (RE) of emotion were unaffected by the intervention. Other researchers found similar results with UE and RE. Nelis et al. (2009) noted respondents’ UE scores did not improve after their training session as was the case in the study by Bucich and Maccann (2019) with RE.

However, as previously mentioned, we are concerned with apparent limitations in our educational material of the AEI skills. Important theoretical aspects of the UE, FE, and RE abilities were poorly addressed. For instance, we over-focused on specific areas in the UE material. We spent too much time on participants acquiring a robust emotional lexicon and understanding the definitions and purposes/triggers of specific emotions. Further, we under-focused on specific dimensions of the FE and RE skills. For the former, content of how mood states enhance/detract in problem solving was missing and needs inclusion; for the latter, the importance of discerning meta-emotions in efforts to self-regulate was minimal and needs to be added (Salovey et al. 2008).

### 4.2. Applications

From the promising and preliminary findings in our feasibility study, we imagine a few real-world applications emerging after the developmental process of our intervention had been completed (Orsmond and Cohn 2015). First, from a programmatic vantage, professors teaching traditional freshmen courses or FYE courses can easily insert this study’s minimalistic procedure into their schedule for the semester (Adamo 2016; Walsh-Portillo 2011). The PowerPoint content, discussion, and quiz require no more than 55 min. Students complete the MSCEIT on their own time requiring approximately 40–50 min for each administration.

FYE Coordinators who prepare fellow professors for such classes can include this study’s intervention in the faculty training. The simplistic, economical, and practical benefits of our procedure might hone the intra- and interpersonal awareness of these educators; enhance the quality and effectiveness of their future presentation of EI information (i.e., our intervention via PowerPoint slides) to their FYE undergraduates; and enrich interactions with students in their FYE class. The study by Lillis (2011) highlighted the relationship between faculty’s EI and the attrition thoughts of their advisees. His findings broaden our scope; enhancement of freshmen’s EI needs to be concomitant with the upgrading of faculty advisor’s EI.

Furthermore, some professors/instructors of traditional freshmen classes or FYE courses may not consider themselves EI savvy. If so, a buddy system could be developed. Faculty with robust EI scores and EI self-efficacy can pair-up with educators that are less confident. Possibly, the former volunteer to present this study’s intervention for some of their colleagues. This may help maintain a high level of instructional quality across all of the freshmen general education and FYE classes in a given semester.

Second, as previously mentioned, robust EI levels potentially present more prospects for freshmen to thrive in their first year in a university and successfully navigate the many challenges in the transition from home and high school (e.g., belonging, stress, anxiety) (Extremera and Fernandez-Berrocal 2006; Thomas et al. 2019; Walton 2018). First-year college students who become emotionally intelligent have honed information-processing abilities that can help them grasp and translate the emotional complexities attached to the aforementioned academic worries and mental health struggles. For instance, adeptness with perception of emotion (PE) empowers undergraduates to correctly identify their emotions that can lead them to being able to better use, understand, and regulate their feelings (Caruso and Salovey 2004). This benefits students’ discerning efforts about their academic ability and purpose at the university. It can also enable them to read others more accurately, a perquisite when trying to fit in and not become socially isolated.

Proficiency in regulation of emotion (RE) can empower them to discriminate their moods and feelings when ruminating on the previously mentioned psychosocial/academic concerns and grappling with the shame from the exposure of personal limits (e.g., poor study habits and high neuroticism) (Mayer et al. 2016; Stein 2009). This discernment can help them get into an adaptive mood and open up thinking that is creative, empathic, and visionary (Caruso and Salovey 2004). In addition, higher understanding of emotion (UE) scores can assist freshmen in recognizing how emotions blend and transition. This affords them the ability to anticipate how they or others may respond to particular circumstances (Caruso and Salovey 2004). Further, high levels of UE may enable them to discern and distinguish cultural variations in emotive expressions among new university peers potentially enhancing a sense of belonging and ability to foster social connections (Mayer et al. 2016).

### 4.3. Limitations and Future Research

The present study was not without limitations. Generalizations derived from the findings are limited to populations with similar characteristics of the sample. With a quasi-experimental one-group pretest–posttest design, we attempted to restrain some of the extraneous variables as the expediency of the ultra-brief EI programming was assessed. To hinder history and maturation effects, we set a one-week limitation between both MSCEIT administrations, one week after the pretest and after the posttest (Wang and Morgan 2010). To curb experimenter/instrumentation effects, a computer scored participants’ answers on the performance-based online assessment of AEI (Knapp 2016). Still, the absence of a control group encumbers the validity of our findings (Kimport and Hartzell 2015). Moreover, the small effect size of facilitation of emotion and the non-enhancement of understanding and regulation of emotion exposes the previously mentioned gaps in the intervention employed in this study. Other training content (e.g., emotional blends and transitions) may increase the effect of the intervention. Such additional information would require editing of the original training material to keep duration at the ultra-brief timeframe.

These limits notwithstanding, the present feasibility study had demonstrable success. Tentative evidence exists that an ultra-brief intervention as a means to educate freshmen on the full-set of the AEI skills can work. Changes in EI scores transpired under these circumstances, just not across all of the skills. Moreover, we have become aware of apparent gaps in the educational material requiring attention. Hence, the importance of an ultra-brief EI enhancement should proceed as a research priority. Incoming freshmen need many resources to adapt in a successful manner to the collegiate environment.

## 5. Conclusions

Our feasibility study tested a novel ultra-brief intervention targeting freshman. Two of four outcome measures improved. From an assessment of this project via the five objectives suggested by Orsmond and Cohn (2015), several modifications to the research design, recruitment efforts, the intervention, and outcome measures were identified and discussed. Further, we processed possible implications of the findings and imagined possible future real-world applications.

The plethora of EI enhancement programs noted in the research literature underscore how people can be empowered to process emotional data in a more efficient manner for adaptive purposes (Puffer 2011). Providing opportunity for undergraduates, particularly freshmen, to experience an EI improvement program aids not only in their transition to college, but also in the kind of the lives they will lead while a student and possibly beyond.

## Figures and Tables

**Table 1:** Correlations between Age and EI Pre-Posttest Variables.

	PE a	FE a	UE a	RE a	PE b	FE b	UE b	RE b
**Age**	.08	−.07	−.01	−.12	.10	−.07	−.07	−.06
***p***	.50	.53	.92	.31	.42	.53	.54	.59

Note: N = 75; PE = Perception of emotion; FE = Facilitation of emotion; UE = Understanding of emotion; RE = Regulation of emotion; a = pretest; b = post-test; *p* = probability of the statistic under the null hypothesis.

**Table 2:** Comparison of Emotional Intelligence Scores between Male and Female Undergraduates.

	PE a	FE a	UE a	RE a	PE b	FE b	UE b	RE b
**Mean** **SD Male**	104.24	116.52	112.50	116.06	110.97	120.58	114.19	120.48
	17.16 *	29.31	18.34	25.45	20.17	29.39	20.05	28.81 *
**Mean** **SD Female**	99.48	107.58	110.75	113.42	106.38	113.49	110.67	113.56
	11.68 *	22.38	17.47	21.21	13.76	23.88	18.71	20.93 *
**T**	1.35	1.49	.42	.49	1.14	1.15	.78	1.15
***p***	.18	.14	.68	.63	.26	.25	.44	.26

Note: N = 32 for males; N = 43 for females; PE = Perception of emotion; FE = Facilitation of emotion; UE = Understanding of emotion; RE = Regulation of emotion; a = pretest; b = posttest; T = t-value; *p* = probability of the statistic under the null hypothesis; and * = unequal variance according to Levene’s Test for Equality of Variances.

**Table 3:** Comparisons of Emotional Intelligence Scores across Academic Majors.

	Source	SS	DF	MS	F	Sig.
**PE a**	Between groups	332.017	3	110.672	.499	.685
	Within groups	9767.415	44	221.987		
	Total	10,099.433	47			
**FE a**	Between groups	1434.232	3	478.077	.631	.599
	Within groups	32,579.020	43	757.652		
	Total	34,013.252	46			
**UE a**	Between groups	916.149	3	305.383	.949	.425
	Within groups	14,159.576	44	321.809		
	Total	15,075.725	47			
**RE a**	Between groups	467.089	3	155.696	.279	.840
	Within groups	24,565.268	44	558.302		
	Total	25,032.357	47			
**PE b**	Between groups	218.273	3	72.758	.201	.895
	Within groups	15,927.045	44	361.978		
	Total	16,145.318	47			
**FE b**	Between groups	276.689	3	92.230	.096	.962
	Within groups	42,274.622	44	960.787		
	Total	42,551.311	47			
**UE b**	Between groups	409.479	3	136.493	.376	.771
	Within groups	15,973.118	44	363.025		
	Total	16,382.597	47			
**RE b**	Between groups	241.769	3	80.590	.117	.949
	Within groups	30,196.699	44	686.289		
	Total	30,438.468	47			

Note: N = 48 (64% of 75 freshmen); PE = Perception of emotion; FE = Facilitation of emotion; UE = Understanding of emotion; RE = Regulation of emotion; a = pretest; b = posttest; Source = source of variation; SS = sum of squares; DF = degrees of freedom; MS = mean Squares; F = f ratio; and Sig. = probability of the statistic under the null hypothesis.

**Table 4:** Descriptive Statistics for the Variables of AEI.

Variable	Pretest M (SD)	Posttest M (SD)
**PE**	101.51 (14.37)	108.30 (16.82)
**FE**	111.44 (25.81)	116.83 (26.46)
**UE**	111.50 (17.75)	112.17 (19.24)
**RE**	114.5 (22.99)	116.51 (26.66)

Note: N = 75; AEI = ability emotional intelligence; PE = Perception of emotion; FE = Facilitation of emotion; UE = Understanding of emotion; and RE = Regulation of emotion.

**Table 5:** Multivariate and Univariate Analyses of Variance for Pre/Post Emotional Intelligence Means and Effect Sizes.

			ANOVA			
		MANOVA	Perception	Facilitation	Understanding	Regulation
	Variable	F(4, 70)	F(1, 73)	F(1, 73)	F(1, 73)	F(1, 73)
		5.52	21.88	4.15	0.29	1.36 *
	TIME	*p* < .001	*p* < .001	*p* = .045	*p* = .59	*p* = .25
		.24	.23	.05	.00	.02
p. eta^2^		(large)	(large)	(small)	(ns)	(ns)

Note: N = 75; Multivariate F ratios were generated from Pillai’s statistic; MANOVA = multivariate analysis of variance; ANOVA = univariate analysis of variance; ns = not statistically significant; p. eta^2^ = partial eta squared marking effect sizes using the range suggested by Cohen (1992), and * = the pretest and posttest means were transformed.

## Data Availability

The data supporting the reported results in this study has been publicly archived with MPDI and can be found at Supplementary Materials.

## Supplementary Materials

The dataset (e.g., demographic information, emotional intelligence pre- and posttest scores) from which the reported results in this study were derived has been publicly archived with MPDI and can be found at (https://www.mdpi.com/article/10.3390/jintelligence9030036/s1).
